# The Experiences of Mid-career and Seasoned Orchestral Musicians in the UK During the First COVID-19 Lockdown

**DOI:** 10.3389/fpsyg.2021.645967

**Published:** 2021-04-09

**Authors:** Susanna Cohen, Jane Ginsborg

**Affiliations:** ^1^Interdisciplinary Department of Social Sciences, Bar Ilan University, Ramat Gan, Israel; ^2^Centre for Music Performance Research, Royal Northern College of Music, Manchester, United Kingdom

**Keywords:** coronavirus, music performance, freelance orchestral musician, sensemaking, self-employed, career, identity

## Abstract

The introduction of social distancing, as part of efforts to try and curb the spread of the COVID-19 pandemic, has brought about drastic disruption to the world of the performing arts. In the UK the majority of professional orchestral musicians are freelance and therefore self-employed. These players, previously engaged in enjoyable, busy, successful, portfolio careers, are currently unable to earn a living carrying out their everyday work of performing music, and their future working lives are surrounded by great uncertainty. The aim of the present study was to examine how established professional musicians are experiencing this period, and to look for similarities and differences between the experiences of musicians in the middle of their performing careers (aged 35–45), with those of older players (aged 53 and over). Single semi-structured interviews were carried out over Zoom with 24 freelance, self-employed orchestral musicians; 12 mid-career musicians aged 35–45, and 12 seasoned musicians aged 53 and over. Thematic analysis identified themes common to both groups: the loss of a much-loved performing career, missing music making and colleagues, and anxiety about the future of the music profession. It also identified differences between the two groups: challenges to their identity as a musician, the extent of their anxiety about finances, the extent of their emotional distress, attitudes toward practicing and engaging in collaborative music making, and confusion over future career plans. Findings are discussed with reference to lifespan models of musicians' career development, the PERMA model of wellbeing, and the concept of resilience.

## Introduction

On March 23, 2020, as part of emergency measures to maintain social distancing and curb the spread of the COVID-19 pandemic, the UK Prime Minister Boris Johnson announced “From this evening I must give the British people a very simple instruction—you must stay at home” (UK Government, 2020). This announcement marked the official start of a national lockdown and brought immediate and drastic disruption to the UK performing arts industry with the cancellation of nearly all live performing arts events, leaving previously successful performing artists suddenly unable to earn a living from their work. Prior to the sudden arrival of the COVID-19 pandemic, the UK music industry was internationally respected and estimated to contribute £5.8 billion annually to the UK economy, with approximately 200,000 people earning their living from working full-time in music (Music UK, 2020), including approximately 14,000 professional orchestral musicians (Association of British Orchestras (ABO), 2019).

The orchestral profession is very competitive (Williamon, 2004; Bennett, 2009; Spahn et al., 2016), with studies showing that classical musicians are highly motivated (McPherson and Zimmerman, 2011; McPherson et al., 2019), investing thousands of hours in perfecting and maintaining their performing skills (Ericsson et al., 1993). Despite evidence indicating that classical musicians face an increased risk of mental health issues (Kegelaers et al., 2020) arising from the potential threat of music performance anxiety (Kenny et al., 2014), playing-related musculoskeletal disorders (Kok et al., 2016), and the stress of touring and unsociable work hours (Steptoe, 1989), other studies suggest that orchestral musicians enjoy their work (Brodsky, 2006; Voltmer et al., 2012) and have high levels of wellbeing (Ascenso et al., 2017), job satisfaction (Brodsky, 2006; Gembris et al., 2018) and life satisfaction (Bonneville-Roussy et al., 2011). Professional musicians tend to be emotionally committed to and passionate about their performing careers (Bonneville-Roussy and Vallerand, 2020), and their self-esteem and identity are often deeply bound up with their work (Dobson, 2010; Teague and Smith, 2015; Ascenso et al., 2017), so that performing becomes a way of life rather than merely a way of earning a living (Oakland et al., 2012).

Orchestral musicians have been shown to have great professional longevity (Allmendinger et al., 1996; Brodsky, 2011). There are few studies examining the careers of professional classical musicians from a lifespan perspective, and existing studies suggest that an adult professional musician's working life can be divided into three different stages (Manturzewska, 1990; Bennett and Hennekam, 2018; López-Íñiguez and Bennett, 2020): (1) the early career stage (approximately ages 20–30, with fewer than ten years of professional experience) characterized by the challenge of establishing oneself in the profession and the search for employment, (2) the mid-career stage (approximately ages 30–45, with 10–25 years of professional experience) described as “the time of greatest performing activity, the widest geographical span of journeys and the highest artistic output. It is a stage of artistic and professional expansion, and of the greatest achievements” (Manturzewska, 1990, p. 135), also characterized by the challenge of consolidating one's professional achievements and the difficulty of obtaining a work/family balance (Teague and Smith, 2015), and (3) the late career stage (approximately age 55 and over, with 25 or more years of professional experience) when there is a greater focus on teaching, acknowledging one's achievements, a transfer of skills, a greater sense of social responsibility and the challenge of managing the potential decline in performing ability due to the aging process (Gembris et al., 2018).

In the UK, unlike most European countries, the majority (over 85%) of orchestral musicians are not salaried, but self-employed as members of freelance orchestras, on short-term contracts as extra or deputy players, or on an *ad hoc* basis (Teague and Smith, 2015; Association of British Orchestras (ABO), 2019; Willis et al., 2019). These freelance musicians build busy portfolio careers comprising a rich and varied combination of performing activities, such as orchestral and chamber music, West End shows, community work, commercial and recording sessions, and also teaching (Bartleet et al., 2019). Maintaining a successful portfolio career as a freelance musician requires a broad skill-set, including effective problem-solving skills, good communication, teamwork, adaptability, and small business and entrepreneurial skills, in addition to excellent performing ability (Bennett and Hennekam, 2018; Willis et al., 2019). Although there are studies showing that self-employment is associated with higher job and life satisfaction than fixed employment by one or more organizations (Warr, 2018), studies of freelance classical musicians have found that work and financial insecurity are associated with increased anxiety (Dobson, 2010; Oakland et al., 2012).

The current period of lockdown has left many freelance musicians in a position of great career uncertainty, with many musicians finding themselves with no work and ineligible for the UK Government's Self-Employed Income Support Scheme (SEISS; Musicians' Union (MU), 2020). Musicians tend to be deeply emotionally invested in their work (Dobson, 2010; Bonneville-Roussy and Vallerand, 2020), and can experience sudden, involuntary career transitions as highly traumatic (Maitlis, 2009; Oakland et al., 2012; Hennekam and Bennett, 2016). In a recent online survey examining the working patterns, income, and wellbeing of performing arts professionals in the UK during the first lockdown, respondents reported a substantial loss of work and income, and an increase in anxiety (Spiro et al., 2021), suggesting that freelance classical orchestra musicians are likely to be particularly vulnerable to threats to their wellbeing during this period. This is all the more so in the light of the growing body of research indicating that the COVID-19 pandemic is associated with significant increases in mental distress, depression and anxiety, generally (British Medical Association (BMA), 2020; Pierce et al., 2020; Tull et al., 2020; Wang et al., 2020).

Lifespan models of professional musicians' working lives (Manturzewska, 1990; Bennett and Hennekam, 2018) suggest that there may be a shift in professional musicians' careers such that their focus, mid-career, on their own performing achievements is replaced over time with an interest in teaching and a sense of social responsibility. It might be, therefore, that musicians at different stages of their professional careers experience this challenging period in different ways.

The present study follows up a previous study of mid-career freelance professional orchestral musicians (Author, submitted). It had two principal aims: (1) to examine how established freelance orchestral musicians at different stages of their careers were experiencing this period of professional uncertainty, and (2) to examine the similarities and differences between the experiences of younger orchestral musicians (aged 35–45) with those of older (“seasoned”) musicians (aged 53 and over).

## Method

### Participants

Participants were 24 professional freelance orchestral musicians, 14 women and 10 men. Twelve were mid-career, aged 35–45 (M = 40.75, SD = 3.96) and 12 were seasoned, aged 53 and over (*M* = 60.50, *SD* = 6.73), with an overall age range of 35–73 (*M* = 50.62, *SD* = 11.44). The mid-career group had between 12 and 23 years (*M* = 17.92, *SD* = 3.68) and the seasoned group had between 32 and 50 years (*M* = 36.5, *SD* = 6.32) of professional playing experience, with an overall average of 27.21 years (*SD* = 10.75). Fourteen of the participants lived with a spouse or partner, seven lived on their own, one lived with a flatmate, one lived with her children, and one was separated and temporarily living with their parents. Twelve participants had children under the age of 18 or adult children who were still financially dependent and living at home. There were 14 string, six woodwind and four brass players (see Table 1 for participants' demographic information). To allow comparison of the findings of the present study with those reported in the existing literature on musicians' careers from a lifespan perspective, the inclusion criteria were ages 35–45 with a minimum of ten years of professional playing experience for mid-career participants, and ages 50 and older with a minimum of 25 years of professional playing experience for inclusion in the seasoned group (Manturzewska, 1990; Bennett and Hennekam, 2018). As the focus was on examining the impact of COVID-19 on musicians who were already well-established and had enjoyed successful freelance careers prior to the start of the pandemic, early-career musicians were not included in this study. All participants needed to be registered as self-employed, based within an hour's drive of London (to increase the homogeneity of the participants' freelance careers prior to the start of the pandemic), and to have earned at least two-thirds of their income from music performance before the pandemic began.

**Table 1:** Summary of participant information.

**Career stage**	**Participant**	**Instrument family**	**Age range(years)**	**Professional playing (years)**	**Marital status**	**Children at home/dependants**
Mid-career	M1	Brass	35–40	15	Partnered	-
	M2	Strings	35–40	16	Single	-
	M3	Woodwind	41–45	21	Married	1
	M4	Brass	41–45	17	Married	2
	M5	Strings	41–45	20	Married	2
	M6	Woodwind	41–45	22	Single	-
	M7	Brass	41–45	23	Married	4
	M8	Strings	41–45	21	Single	-
	M9	Woodwind	35–40	12	Single	-
	M10	Strings	35–40	18	Married	1
	M11	Strings	35–40	12	Separated	-
	M12	Strings	41–45	18	Married	1
Late career	S1	Strings	56–60	35	Married	1
	S2	Strings	66 +	40	Married	-
	S3	Strings	61–65	33	Married	2
	S4	Strings	51–55	33	Married	2
	S5	Woodwind	66 +	50	Divorced	-
	S6	Strings	51–55	32	Single	-
	S7	Woodwind	66 +	35	Single	-
	S8	Woodwind	66 +	48	Married	-
	S9	Strings	51–55	30	Married	3
	S10	Brass	51–55	33	Divorced	2
	S11	Strings	56–60	34	Married	1
	S12	Strings	56–60	35	Partnered	-

*M, mid-career musician; S, seasoned musician*.

### Procedure and Data Collection

Once the study had received ethical approval from the researchers' host institution, the participants were recruited from notices posted on Facebook pages for the groups Coronavirus Crisis—Musicians' Information Page and Musicians' Virus Forum (UK), and via snowball sampling. Potential participants were asked to make contact with the first author via Facebook Messenger or email to confirm that they met the inclusion criteria. They were then sent the participant information sheet via email, and a mutually convenient time was set up to carry out the interview on Zoom. At the start of the Zoom interview, participants gave verbal consent and either signed electronically, or printed out and signed hard copies of consent forms which they returned to the first author via email. The interviews lasted approximately one hour and were recorded on Zoom. Immediately after the interview the video recording was deleted and the audio recording was saved on the first author's password-locked computer. It was decided to carry out the interviews on Zoom as this platform enabled the closest approximation to a face-to-face interview during a time of lockdown, facilitating the possibility of real-time auditory and visual interactions (Marhefka et al., 2020), as well as allowing the use of the share-screen function for providing informed consent. A semi-structured interview format was chosen so that the topics discussed could be limited and focused, while providing sufficient flexibility to follow up particular points of interest. The interview was divided into two main sections. The first consisted of questions about the participants' careers prior to the pandemic, and the second focused on the impact of COVID-19 on the lives of the participants (see Appendix A in Supplementary Material for interview guide). This provided a structure within which the participants could reflect on the ways in which the pandemic had changed their lives. Participants were asked at the end of the interview if they would be willing to take part in a follow-up interview in a year's time. The interviews were carried out in May and June 2020.

### Data Analysis

The aim of this qualitative study was to understand how freelance professional orchestral musicians were experiencing the impact of COVID-19 on their lives. A social constructionist approach (DeLamater and Hyde, 1998) was taken such that the knowledge generated was understood to have been constructed in the interactions between each participant and the interviewer. The transcripts were analyzed using thematic analysis, an inductive, bottom-up, recursive method that is useful for examining the perspectives and experiences of multiple participants, identifying patterns of meaning across data, highlighting similarities and differences between participants and generating unanticipated insights (Braun and Clarke, 2006, 2019), following the steps outlined by Braun and Clarke (2006). First, all 24 transcripts were read through several times by the first author to familiarize herself with the data. Second, the transcripts were coded for meaningful phrases within and between the two groups (mid-career and seasoned musicians). Third, the codes were grouped into themes characterizing the impact of COVID-19 on musicians' lives. Fourth, the themes were grouped into overarching themes and the similarities and differences between themes arising from data provided by the mid-career and seasoned musicians respectively were examined. Fifth, all codes, themes, overarching themes, and the similarities and differences between the data from the two groups of musicians were reviewed. To ensure the credibility of the findings, the second author read through nine of the transcripts (interviews with six mid-career and three seasoned musicians), and analyzed three of them (interviews with two mid-career musicians and one seasoned musician) independently. The codes, themes, and overarching themes identified in these transcripts were then discussed, reviewed and agreed upon. This provided a richer and more nuanced reading of the data (Braun and Clarke, 2019), had only one researcher carried out the analysis, and ensured that the criterion of trustworthiness was met (Lincoln and Guba, 1986).

## Results

**Figure 1:**
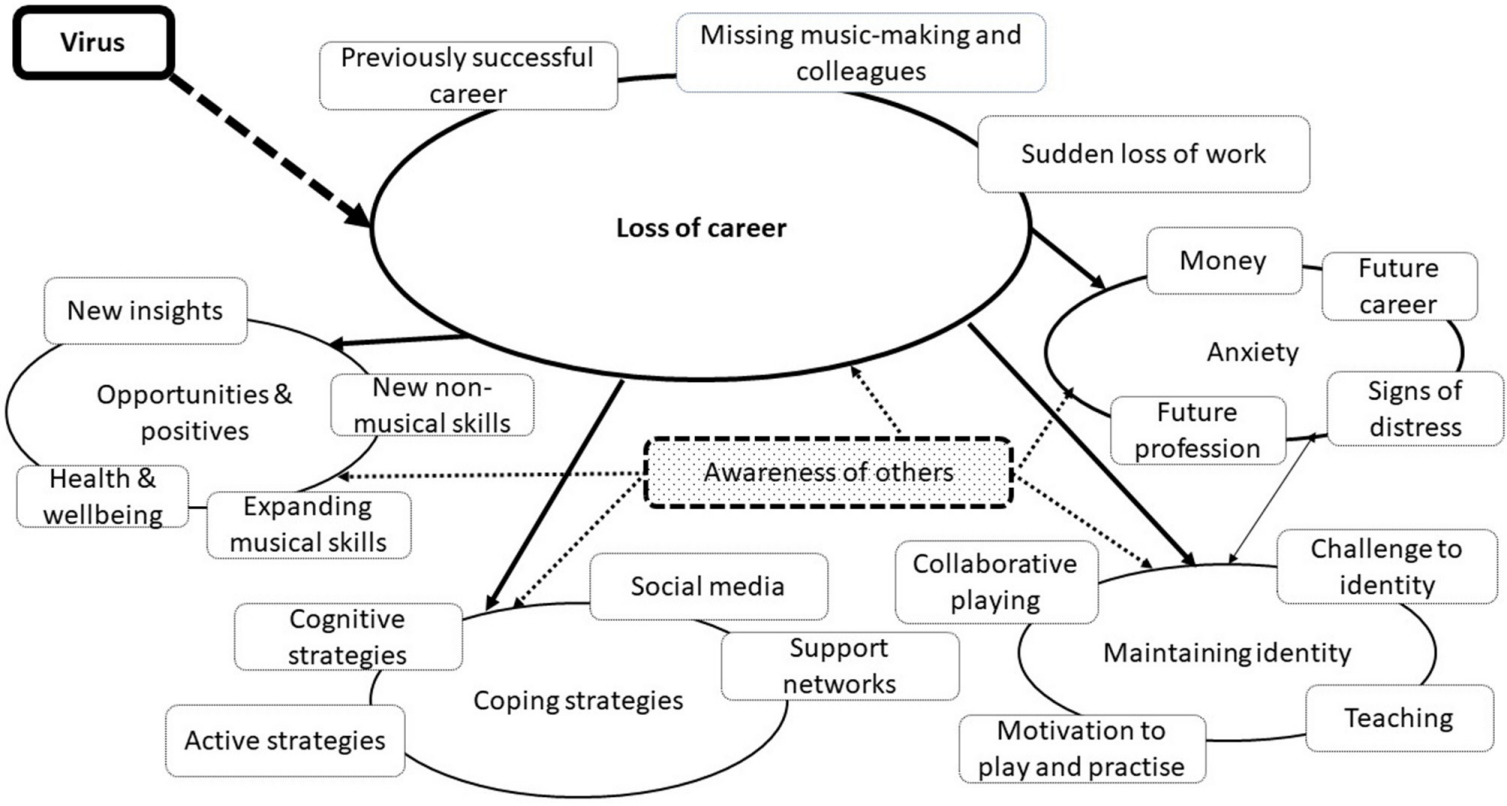
Model of themes and overarching themes.

The thematic analysis produced a final set of one higher-level (central) and four lower-order overarching themes, comprising 19 sub-themes, and there were two additional themes that were not encompassed by the overarching themes. Loss of career was identified as the central overarching theme, and Anxiety, Maintaining Identity as a Musician, Strategies for Coping, and Positives and Opportunities were identified as lower-order overarching themes. Being Ill with the Virus and Awareness of Others were identified as additional themes, the latter a latent theme that was not referred to explicitly by participants but was identified during the analysis of the data. A model providing a graphical representation of the associations between the overarching themes and sub-themes is presented in Figure 1.

A summary of the overarching themes, sub-themes, and number of participants contributing to each sub-theme in the mid-career (M) and seasoned (S) groups is presented in Table 2. The number of participants contributing to each sub-theme is reported, rather than the number of times each sub-theme was identified, to provide a measure of the prevalence of the qualitative findings and facilitate an examination of similarities and differences between the two groups.

**Table 2:** Overarching themes, sub-themes, group and number of participants for each sub-theme.

**Overarching theme**	**Sub-themes**	***N* (total)**	**M**	**S**
1. Loss of career	1.1 Previously successful career	24	12	12
	1.2 Sudden loss of work	24	12	12
	1.3 Missing music making and colleagues	24	12	12
2. Anxiety	2.1 Anxiety about money	22	12	10
	2.2 Anxiety about individual future careers	24	12	12
	2.3 Anxiety about future of classical music profession	24	12	12
	2.4 Emotional and behavioral signs of distress	14	9	5
3. Maintaining identity as a musician	3.1 Motivation to play and practice	22	11	11
	3.2 Challenge to identity	12	11	1
	3.3 Collaborative playing	15	6	9
	3.4 Teaching	21	11	10
4. Strategies for coping	4.1 Support networks	22	11	11
	4.2 Cognitive strategies	21	9	12
	4.3 Active strategies	18	6	12
	4.4 Social media	14	9	5
5. Opportunities and positives	5.1 Expanding musical skills	12	4	8
	5.2 New non-musical skills	2	2	0
	5.3 Health and wellbeing	16	7	9
	5.4 New insights	8	5	3
6. Being ill with the virus		12	4	8
7. Awareness of others		23	11	12

*N, total number of participants mentioning theme; M, number of mid-career participants; S, number of seasoned career participants*.

### Loss of Career

Loss of Career was identified as the central overarching theme, linked to the four lower-order overarching themes and the two additional themes. Loss of career encompassed three sub-themes; *previously successful career* prior to the start of the COVID-19 pandemic (sub-theme 1.1), *sudden loss of work* with the start of the lockdown (sub-theme 1.2) and *missing music making and colleagues* (sub-theme 1.3). All 24 participants reported having much loved, *previously successful careers* (sub-theme 1.1) prior to the COVID-19 pandemic, saying, for example, “I love freelancing! I think it's the, the best way to be an orchestral musician actually… it's lovely because I can drop in and drop out and, and have this wider circle of friends as a consequence” (M6), and “I'm very busy, I earn well and it's, and I wouldn't have it any other way… It's the diversity, it's the camaraderie, it's the income” (S9).

All 24 participants described experiencing a *sudden loss of work* (sub-theme 1.2) at the start of the lockdown. One said

Life as I knew it, completely stopped. Well, I mean basically, my diary's disappeared overnight, all gone! It's like, it's like I've died or something! You look at your diary and you think “was this the diary of somebody who just dropped dead?!” Suddenly a very, very busy and supposedly successful life has just evaporated, there's just nothing there, nothing left. (S6)

Another said, “Yeah, I started off the year thinking well financially I'm going to be absolutely fine this year, and… yes, so it all disappeared in 24 hours really, it was extraordinary” (M6). The sudden loss of work was experienced as traumatic for many of the participants, according to Tedeschi and Calhoun's (2004) definition of traumatic events as those in which one's fundamental assumptions about the world are severely challenged by intensely negative and unexpected events, and was experienced as emotionally overwhelming (Van der Kolk and Fisher, 1995) by one-third of the participants (M = 6, S = 2): “It suddenly just felt like the rug had [been] pulled out from under my feet, and I felt as if I was grieving my old life, I felt, I felt like it was, I was in the middle of a bereavement, I felt, it did feel, it felt like a death, because it was so sudden… I felt like I was sort of in freefall… I just didn't quite know what to do with myself” (M8). Another participant described the last concert they had played in before lockdown as follows: “At the end of that concert, it was a terrible feeling, it felt like, it felt like the analogy of the lights going off all over Europe, it felt like the beginning of a war… sorry I might get a bit emotional talking, yeah (silence), sorry, ahh, right, yeah, it's when I talk about music that I get most upset really (weeps), sorry” (S1).

All 24 participants described how much they were *missing music making and colleagues* (sub-theme 1.3):

I miss the music, I miss sitting in a X section, I miss driving to work or whatever, turning up, going to the canteen, getting the coffees, getting ready, going in, warming up, looking at the part a bit, and just yeah, and the sounds and sights of an orchestra getting ready and people coming in and saying good morning, just the whole thing that surrounds it as well. That's what I love, that's why I do it, and the music itself, the great wonderful music … being surrounded by that sound and to play with a fantastic orchestra like X is an honor and a privilege. (S1)

Another participant commented “I miss playing with people, but I (weeps), sorry… well for me it's, it's a lot of, it's a lot of, it's a big emotional outlet, the music, and playing it with people… just makes a huge difference in life” (M1), illustrating the point that playing is an important means of emotional expression for many players (Simoens and Tervaniemi, 2013). The data contributing to this first overarching theme thus support existing evidence that musicians love their work (Brodsky, 2006), and enjoyed high levels of job satisfaction (Gembris et al., 2018) and wellbeing (Ascenso et al., 2017) prior to the start of the pandemic.

### Anxiety

The second overarching theme, Anxiety, was identified in all of the interviews, with participants describing *anxiety about money* (2.1), *anxiety about individual future careers* (2.2), *anxiety about the future of the classical music profession* (2.3) as well as reporting *emotional and behavioral signs of distress* (2.4), stemming from the sudden loss of career. All participants in the mid-career group and ten in the seasoned group reported *anxiety about money* (2.1). Twenty-one participants (M = 12, S = 9) had applied for the UK Government's SEISS, a scheme introduced with the aim of providing self-employed workers with the same level of support as that being paid to employed workers through the UK Government's Coronavirus Job Retention Scheme (UK Parliament, 2020) and intended to provide 80% of average monthly trading profit. Thirteen of the 21 participants (M = 8, S = 5) received the government grants, but eight (M = 4, S = 4) were ineligible: five (M = 2, S = 3) because their PAYE earnings were slightly higher than their self-employed profits for the three years in question. One seasoned participant had earned just over the £50,000 threshold for receiving the grant, one mid-career participant had spent one of the three years in question working abroad, and one mid-career participant was a foreign national who had lived and worked in the UK for more than ten years. This participant had applied for Universal Credit, despite understanding that this would endanger their right to remain in the UK after Brexit. The finding that eight of the 21 participants (38%) who applied for the SEISS were ineligible reflects the findings of a large-scale Musicians' Union survey which found that 33% of self-employed musicians were ineligible for the SEISS (Musicians' Union (MU), 2020).

The 12 married participants were all married to other musicians (M = 6, S = 6), and in one case both partners had fallen through the gaps for government support, leaving their family in great financial distress: “You know, we're, we're selling, we've sold lots of stuff, because we have virtually, our household income is maybe 15% of what it was previously, erm and you can't just suddenly function on that, you know… we sold our caravan” (M5). All 12 of the mid-career participants and two of the seasoned participants were anxious about the imminent likelihood of acute financial distress: “I'm going to have to, I'm going to have to try and work out what to do now, in order to have a roof over my head, and be able to eat” (M8). In contrast, nine of the seasoned participants commented that they felt lucky to be financially more established and secure than they thought younger less established players were likely to be: “I'm, I'm OK. I'm totally afloat and I've saved some money, so, again, that's not true for the youngsters” (S6). Of the three seasoned participants who did not apply for the SEISS scheme, two were over the age of retirement and receiving a pension so decided not to apply, and one had traveled abroad in order to spend this period with their elderly parents who were not resident in the UK.

All 24 participants expressed *anxiety about their future careers* (sub-theme 2.2) and nearly all of the mid-career participants (M = 11) described the difficulty of coping with the uncertainty surrounding their careers: “I don't have the safety net of a job in an orchestra so someone in the management can worry about it… now it's slightly sort of, like a black hole of uncertainty really, no matter how much you'd like to throw at it, there are questions you can't answer” (M12), and “I, I don't know that it will be all right for me, and I am not just starting out, I am quite high up in the rankings of my instrument as it were, and I, most days can't see that it will be all right” (M1). These mid-career participants (M = 11) also expressed confusion and distress over what they thought they should be doing at this time, with nine of the mid-career participants saying that they needed career advice:

I feel like I've done this all my life, and I don't know what else I can do, so, I'm feeling really uncertain about everything. I'm nearly 44 and I'm not sure that I'm up for re-training completely to do something else, I'm not sure I've got that in me right now… I need career advice from somebody I guess, but I really don't want to do anything different, but I think we'll probably have to, and I don't really know what, and that's what's upsetting me. (M3)

Another mid-career participant commented “I like going to work, I like working, I love my job, I don't want to not do that… Maybe it's time to diversify and, I'm open to that, but currently I feel that I'm very much on my own, and I need someone to show me how to do all of that stuff” (M8).

Eleven participants were actively considering leaving the profession; seven mid-career participants were contemplating retraining:

You know on a bad day I'm like literally going on the website you know, civil service, going, right, I've got a first class degree, I've got really good qualifications, I'm articulate, I'm well-organized, I'm not, I mean I'm 41, I'm not a spring chicken but nor am I sort of approaching retirement, so do something else, do something else. And then I have this kind of miserable evening at looking at all these jobs and going “well I don't know what any of that is” or “I don't know whether my skills refer to that” and then go “oh maybe I'll be, I'll just go and be a teacher then” and then think, “what, that's poorly paid, stressful, parents are a nightmare, don't want to do that, if I'd wanted to be a teacher I would have done it by now. What about a law conversion? Oh well, you pay for doing that,” so I have all this kind of erratic thinking over, just do something else, just do something else, you could still enjoy playing as a hobby, do something else. (M4)

while four of the oldest seasoned participants were considering that the current crisis might have brought about their enforced, unplanned early retirement: “I personally wouldn't be at all surprised if I never do another concert again… it's been a kind of, well maybe I've retired, maybe I have” (S3).

In the current study, 11 of the 24 (46%) participants were thinking of leaving the profession. This is lower than the 64% reported by ENCORE, an online musicians' booking platform survey of 560 musicians (ENCORE Musicians, 2020), but higher than the Musicians' Union finding of 34% in a survey of 2,000 members (Musicians' Union (MU), 2020). By contrast with the seven mid-career participants who were thinking of retraining outside the field of music, seven seasoned participants and two mid-career participants were considering expanding their musical work to include areas other than performing: “[I want] to rely less on my performing playing, let's say for income, and to start teaching a lot more online, perhaps even setting up a kind of online presence, alongside a few colleagues, that might create something” (S4). This will be discussed further in the context of the fifth overarching theme of Opportunities and Positives.

All 24 participants expressed *anxiety about the future of the music profession* (sub-theme 2.3): “I know they keep talking about the ‘new normal' but if the new normal means that concert halls and theaters can no longer open and operate, that can't be our new normal” (M6). Thirteen (M = 9, S = 4) expressed a sense of injustice and unfairness in the UK government's treatment of self-employed musicians and lack of support for the arts:

My brother's, you know, we earn the same, similar amounts, and he's, he's being offered money [from the orchestra he is employed by] and I'm not, why the difference?… that really upset me… they say “now we're helping the self-employed”, “now everybody's getting the 80%”, but they're so not, people are falling under it… and that discrimination against self-employed is, is really hurtful, when we're all supposed to be in it together. (S9)

Notably, the majority of respondents to Spiro et al.'s (2021) survey of performing arts professionals in the UK also reported loss of work, worry about finances and uncertainty and concern about the future. One participant in the present study talked openly about her frustration and jealousy of those people who she felt were oblivious to the impact of COVID-19 on people's lives: “I find it quite hard to be in touch with people who are not doing the same, who don't have the same jobs as us. I feel very kind of awful to say this, but I just feel very jealous that they still have their livelihoods and it hasn't affected them very much, and they're having quite a nice time actually, some of them” (M3).

Fourteen participants (M = 9, S = 5) reported experiencing *emotional and behavioral signs of anxiety and distress* (sub-theme 2.4). These included disturbed sleeping (M = 5, S = 2) and mood disturbances (M = 7, S = 2):

It feels, I feel like “what's the point?” On a bad day, what's the point of getting up, there's sort of nothing to do. I feel like I don't really have a purpose… getting up first of all would be difficult, and just sort of, just basic looking after yourself, brushing your teeth, you sort of put that off, or have a shower, put that off, or [can't] quite be bothered to cook, so order some rubbish food to be delivered… then feel a bit sort of rubbish about yourself… some days I find it difficult to claw my way out of that. (M8)

All six of the mid-career participants with younger children described moments of great stress within the family during this time when parents were responsible for childcare and home-schooling, supporting recent reports that the COVID-19 pandemic has placed a great strain on the family unit (Spinelli et al., 2020): “It's been hard, my mood has not been great. I have found myself getting cross with my family, just because I feel sometimes, sometimes I feel a certain, you know, sense of resentfulness because I find myself unable to work” (M12). Two seasoned participants mentioned worrying about their older parents getting ill with the coronavirus, and a further two seasoned participants described their own health worries about contracting the coronavirus, including one who had suffered from panic attacks: “I've had a couple of panic attacks … I started sweating a lot, and I thought, you know, “goodness me, are these symptoms coming on,” so I got a bit panicky about that” (S1), supporting the findings of recent studies showing that levels of distress and anxiety have increased during the pandemic (British Medical Association (BMA), 2020; Pierce et al., 2020).

### Maintaining Identity as a Musician

The third overarching theme, maintaining identity as a musician, comprised four sub-themes: *motivation to practice* (3.1), *challenge to identity* (3.2), *collaborative playing* (3.3) and *teaching* (3.4). The subject of *motivation to practice* arose in all of the interviews. The majority of participants (M =11, S = 11) said that they were playing during this period, although their motivations varied. Five participants (M = 3, S = 2) described practicing in order to reconnect with and strengthen their identity as a musician:

I'm playing, doing a bit of kind of scales and exercises every day, it's a bit like, it's kind of like my religion, or my meditation or something. I kind of need to do that every day, to remind myself who I am, to ground myself, so that, if a day goes by when I feel I haven't accomplished very much, and these days are very strange like that, I can at least go “well I did my bit of playing.” (S2)

Twice as many seasoned as mid-career participants said they were practicing to improve their skills (M = 4, S = 8), with mainly seasoned participants commenting that they were enjoying practicing during this period of lockdown: “I love the, I'm disciplined about practice, and I'm just doing sort of a couple of hours and going, doing practice for practice's sake, and that's a lovely, that's been a real bonus from COVID” (S9), supporting existing evidence that professional classical musicians have high levels of intrinsic motivation (Appelgren et al., 2019). More seasoned than mid-career participants said that they were practicing to maintain their skills for when playing resumed (M = 3, S = 8): “Basically I need to keep my technique, my technique there, so that if things suddenly do recover, then I'm in a position to carry on working and people won't turn round and think ‘what the heck is he doing?”' (S1); and equal numbers of mid-career and seasoned participants (M = 3, S = 3) said they were practicing so that they would be able to demonstrate to students: “It's those higher-end students in terms of their ability and their focus that are making me make sure that I don't go ‘try it like this' and then it be a cacophonous mess and then they look at me as if to say ‘well I think I won't try it like that,' so that's what's keeping me in practice” (M4).

Of the eight participants (M = 4, S = 4) who talked about the difficulty of motivating themselves to play and practice during this time, three mid-career participants expressed a “What's the point?” feeling: “I think more and more, because of, I think the financial things, I've not really felt like practicing very much” (M9), and one of the mid-career participants described how hard it was to find a moment to practice when she was also looking after her children: “I've got two small children and I have to cook three meals a day and keep the house and you know everything else and I can't really justify just going up to a room to practice my studies, when that's sort of going on” (M5). Three seasoned participants (two players of lower string instruments, one lower woodwind player) described the difficulty of being motivated to play alone, rather than as part of an ensemble: “Our instruments are not meant to be played on their own, we're pack animals, we play as a group” (S8).

Nearly all the mid-career and one of the seasoned participants (M = 11, S = 1) described experiencing this time when they were unable to work as a *challenge to identity* (3.2):

You know I think a lot of musicians they get their identity from what they do … I confess it was difficult for me going into the lockdown after a while, because I had no, no, nowhere to escape to you know, I was just left with my family, and I had this young child that needed me a lot, and erm, and my sense of pride or purpose that came from playing, doing the sort of things that I had been doing was gone. (M12)

One participant described her confusion over the loss of her partner's identity as a musician when he started working in the evening doing food deliveries to make money after his job in a West End show abruptly stopped:

He would always leave the house at 5.30 and drive into London, wearing black clothes, and be back at 11, and do his show and that's been ever since I've known him. And now he's still doing the same, but for a delivery job and leaving a little bit earlier, but he's still wearing black clothes and still coming back at the same time, but for some reason, it's, I'm, I'm struggling with it… I think the thought that we don't, everything we've done amounts to nothing right now. (M3)

Several mid-career participants expressed disappointment in themselves at their lack of motivation to practice during this time:

I've found it very, very difficult to be motivated to do that [practice], very difficult, which is, hard, because I sort of feel a bit disappointed with myself almost, that I haven't been able to, you know, this is the thing I love, as well as my job, it's very much a part of who I am, playing an instrument professionally, and, I, I, I haven't done it… you start to think “what is it I actually do?” (M8)

These examples support previous evidence that musicians' sense of self and identity is deeply bound up in their work (Dobson, 2010; Teague and Smith, 2015), and also that self-employed musicians find it difficult to maintain a sense of identity in the absence of some kind of professional or organizational holding environment (Petriglieri et al., 2019). In contrast, the seasoned musicians did not report these challenges to their identity as musicians: “I'm, I'm still you know, I'm still a [instrument] player, I haven't got any work, but I'm still a [instrument] player” (S3). They also seemed more able to bring a longer-term time perspective to this period: “You really do have to take the rough with the smooth, and that it's, the profession doesn't go in a straight line, life doesn't go in a straight line” (S6).

The majority of participants (M = 6, S = 9) had been involved in some kind of *collaborative playing* (sub-theme 3.3) including participation in online recordings, street concerts and also playing informally with family members. Just over half of the participants had participated in online Zoom clips (M = 5, S = 8) in which they had recorded their individual parts at home, usually onto a mobile phone, while listening to a click-track or basic soundtrack on headphones, and then sent these recordings via email or WhatsApp to a sound engineer who collated the individual parts. Although one might have expected that mid-career players would be more comfortable with technology and more ready to engage in Zoom recordings than seasoned players, more seasoned participants were involved in online recordings than younger participants. One mid-career participant commented: “You know people were like ‘oh, do you fancy recording, you know *We'll meet again*?' ‘No, I don't, no, I do not fancy doing that'… I suppose it's just been like I haven't got time for this, it's just another thing that someone's asking me to do, and I don't want to do it, so I sort of said no” (M4). Every participant who had been involved in collaborative online recordings described them as frustrating and no substitute for playing together: “It wasn't a satisfying way of playing together… it's just, you can tell that there's something not going on and that's the live interaction that happens between musicians in real time. You can't, you just can't replicate that remotely” (S4). However, despite the frustrations, seasoned participants were more positive about and tolerant of Zoom playing opportunities than the mid-career participants: “It's just, it was nice to see all the familiar faces on the screen when you saw the end results and to know that, there's a kind of solidarity and a feeling like, a warmth toward other beings who are all also cooped up at home, and who all have lost their careers right now” (S6). Four participants (M = 2, S = 2) had contributed to other live collaborations, for example playing in street concerts for neighbors, such as *Somewhere over the Rainbow* for the National Health Service (South Wales Guardian, 2020). Two seasoned participants reported enjoying this and found it reinforced their identity as musicians:

[playing with husband] The whole street came out, which was just wonderful and kind of stood around and applauded and wanted it again, and it was very interesting because I was really buoyed up by that for a while, and I thought “oh my gosh!” you know, “performance is what I do” and I'm not doing it at the moment, and this was just a tiny flavor of that, and it really meant something. (S2)

By contrast, the two mid-career participants expressed frustration and found it challenged their identity as a professional musician: “It just feels, it just feels, I feel a bit like, the things that we're doing, are things that we used to do when we were students, and you know like straight out of college” (M3). Seven participants (M = 3, S = 4) had taken part in informal family music making, either with children or older parents, which they described as enjoyable, but not the standard of their normal professional music making.

Nearly all of the participants (M = 11, S = 10) were engaged in instrumental *teaching* (sub-theme 3.4). The mid-career participants talked about appreciating instrumental teaching for the income and for the opportunity to reinforce their identity as musicians: “It's good because I'm up, I'm dressed, I'm washed, I look presentable, I do something and that makes me feel like me again” (M8). The mid-career participants also reported that they had considered teaching second best to performing: “I've always felt slightly erm, that maybe people felt that the really good players earned all their money by playing and not by teaching, and as it is in the current situation, it's the only thing that's, that's saved us” (M5), supporting the suggestion (Bennett, 2009) that teaching is generally considered to be lower in the hierarchy than performance in the freelance musician's portfolio career. In contrast, many seasoned participants commented that teaching was an extremely meaningful and important part of their professional lives: “My teaching is incredibly important to me, that's becoming a bit more of a focus, alongside the playing of the violin, the pedagogic nature of it, is sort of, intrigues me more and more” (S6). The changing role and status of teaching over the span of a musician's portfolio career will be considered further in the Discussion section.

### Coping Strategies

Participants made use of a wide variety of strategies to help them cope with the sudden loss of work, which could be grouped broadly into the sub-themes *support networks* (4.1) of friends, family and colleagues, *cognitive strategies* (4.2), *active strategies* (4.3) and use of *social media* (4.4). The majority of participants (M = 11, S = 11) described the importance of *support networks* (4.1) of friends, family and colleagues: “I keep going back to that support network of friends who are also musicians, but it's, without them, I know it would be, I think I probably would be in a very serious mental health place of concern” (M6). One of the mid-career participants had attended a number of online meetings set up by various musicians' organizations and described how helpful she had found them: “I went to this X Musicians' Zoom… and it was just sort of nice, and that's made me feel better, and feel that we're all in it together, and it's not just me and [partner] battling this whole thing, this kind of sense of musical community” (M3). One mid-career participant described getting support with childcare from her parents via Zoom: “He's [child's grandad] now giving my little boy sort of piano lessons… just to give us time to erm, try and apply for jobs or just admin, or anything that we need to do in the house” (M3), and two seasoned participants described how their older, retired parents had offered to help them financially.

The majority of participants made use of a range of *cognitive strategies* (sub-theme 4.2). These included the use of positive and soothing self-talk (M = 9, S = 12), with the recurrence of phrases such as “everybody's in it together”: “I try and remember that I was busy, I was in the right place, I was getting called for work, and that's not, that element of what I've achieved in my career, it's not just going to vanish, because everybody is in the same boat. It's not like I've taken a year off and everybody else has continued” (M11). Another common strategy, used particularly by mid-career participants, was trying to live in the here and now and not allow yourself to ruminate (M = 9, S = 4):

I'm a worrier by nature, to think about “oh gosh, what's going to happen, what's going to happen, what's going to happen?” and I've been really consciously trying to yank myself out of that every time I catch myself… If I go down that rabbit hole, you know, it feels like the world is ending, that there's no hope, and I just, and I know for me, in order to survive, I can't allow myself to do that. (M8)

However, three mid-career participants also described coping strategies that have been found to be maladaptive (Stallman, 2020) in reducing anxiety: “So my coping mechanism is basically to step back and say maybe that is the end of it for me, you know I feel very lucky to have worked (weeps)… in the end, you know, I'm just drawing, I'm just drawing into a very small world of trying to buy food for as little money as I can, and keep [the] children going without spending any money on anything” (M5).

Nearly all of the seasoned participants but less than half of the mid-career participants (M = 4, S = 10) described reframing this period of their lives as temporary, and expressed positive hopes for the future: “It's not going to be forever… I'll probably look back and think well that was six months of my career that was very odd and a bit shit, but it is only six months” (M11), with the majority of seasoned participants contextualizing this time as finite by describing other challenging times in their professional and personal lives that they had overcome: “Uncertainty's not a problem for me, because I've had a very difficult five years personally, which had a huge amount of uncertainty in it, and difficulty. So lockdown, I know, it's just the same actually (laughs) or maybe slightly easier” (S10). These findings suggest that the seasoned participants may be more emotionally robust than the mid-career players, which will be addressed in the Discussion section. While some seasoned participants framed the current forced stop as a time to acknowledge that they'd had long, successful careers: “I don't feel bereft in any way that I'm not doing it right now, maybe because I've had so many rich years of doing it. I'm not at the start of my career, I've got a lot to show for it” (S6), other seasoned participants reframed this period as an opportunity to stop and maybe consider new paths: “Personally I'm trying to use it as like a gestation period of allowing new ideas and new thoughts and concepts… I was going to have a sabbatical at the end of this year, it feels like the sabbatical's just arrived early” (S6). The topic of new opportunities and approaches will be addressed further in section Opportunities and Positives.

Participants made use of a variety of *active strategies* (sub-theme 4.3) for maintaining health and wellbeing. These included giving the day a structure (M = 6, S = 12): “The whole lockdown situation is just like everything's like nebulous and just there's, there's no, no structure, so you just have to kind of make your own structure, so I'm trying to like allocate certain times” (S7), engaging in physical exercise and being outside (M = 9, S = 7): “I'll just go for a bike ride or go for a walk, remember that there's a world outside that's very, very nice and not everything exists around the violin” (M2), and doing cooking, home maintenance, and gardening (M = 2, S = 7). These are all activities that have been found to be useful for reducing anxiety and facilitating mental health (Stonerock et al., 2015; Meunier et al., 2019). Spiro et al.'s (2021) survey of performing arts professionals also found that increases in physical activity during lockdown were associated with higher wellbeing, highlighting the importance of engaging in physical activity. In the present study, five mid-career participants with young children said that caring for young children had helped to distract them from other anxieties: “They still really need me… having them is such a, such a focus point, you haven't really got that many hours to sit around kind of panicking” (M4). One mid-career participant reported using a mobile phone app to help regulate mood and anxiety and another mid-career participant reported finding prayer helpful.

All of the 14 participants (M = 9, S = 5) who said they used *social media* (sub-theme 4.4) reported having conflicting feelings about it, reflecting recent findings indicating that social media can have both positive and negative influences on affect (Gao et al., 2020). Whereas one participant commented: “I just found it can ruin my whole day reading one thing, and I've taken it off my phone” (M3), another said “I've enjoyed social media during this time, I think it's been one of the like, a real blessing to have it… there's been some caring people out there and some useful articles” (M10).

### Opportunities and Positives

Despite the drastic changes and uncertainty surrounding their working lives, 14 participants (M = 4, S = 10) reported opportunities and positives arising out of this period. These were grouped into four sub-themes: *expanding musical skills* (sub-theme 5.1), *new non-musical skills* (sub-theme 5.2*), health and wellbeing* (sub-theme 5.3) and *new insights* (sub-theme 5.4). Twelve participants (M = 4, S = 8) talked about using this time for improving and *expanding musical skills* (sub-theme 5.1), supporting evidence that musicians are highly intrinsically motivated (Appelgren et al., 2019). It is interesting to note that twice as many seasoned than mid-career participants were occupied with improving and expanding musical skills during this period. Some focused on developing their playing and performance skills: “This opportunity to fix those kind of things, or to at least address them, and try to address them in a, in a, in a successful way, in a slow, determined successful way, as opposed to what's going to get me through this next performance” (S12). Two mid-career participants described how this time had been good for developing online performing opportunities:

Live streaming quartets, you know on Instagram Live, Facebook Live… we've been pretty creative I think. I mean it's been fantastic for us… our profile has been more active in lockdown than it had for a long time previously, just because we were, you know, coming up with initiatives and people were tuning in… The thought, at this time last year, of doing solo recitals, learning a Bach sonata and that sort of thing and doing it on Instagram, I just wouldn't have done all that, so it's been positive, definitely. (M10)

Four of the seasoned participants had used the time to develop non-performance focused music projects including expanding a website selling musical instruments, involvement in a music-science research project, creating an online forum to help string players, and marketing their own recordings. Three participants (M =1, S = 2) had started to experiment with composing, arranging music, and learning to play in new styles. Eleven (M = 3, S = 8) had focused on developing their teaching and community-music work; again, the seasoned participants were more highly represented here than the mid-career participants: “I am consciously skilling myself up, up-skilling myself, and at the same time, up-skilling the professors we have in our department, so that we come out of this with more, more capability, more capacity, more feeling that that we've gained things rather than lost things” (S10). *New non-musical skills* (sub-theme 5.2) included learning a new language and doing voluntary work (M = 2), both taken up by mid-career participants.

Even though the interviews were carried out at a time of great stress and anxiety, 15 participants (M = 6, S = 9) described improvements in their *health and wellbeing* (sub-theme 5.3). This was attributed, in part, to exercising more and drinking less, supporting the findings of studies that musicians generally tend to have sedentary lifestyles and above-average alcohol consumption (Vaag et al., 2016). Just under half the participants (M = 3, S = 8) mentioned being less anxious and more relaxed, supporting evidence that stress and perfectionism can be an integral part of orchestral life (Kenny et al., 2014, 2018; Vervainioti and Alexopoulos, 2015). One reported, “I feel funnily enough, I feel a lot more relaxed in myself on this lockdown time. I'm not, I'm not stressing about [the diary service] coming off my phone thinking ‘oh what's that work' or ‘my whole life is clashing again”' (M11), and another player commented “It's amazing when the phone doesn't go, you know it's because you haven't fucked up, it's because the phone can't go” (S9). Notably, most of these participants reporting less anxiety and feeling more relaxed were in the seasoned group, and all three mid-career participants and five of the eight seasoned participants had received government financial aid, suggesting that this may have helped to alleviate some of the stress of financial hardship, found to be related to lower wellbeing in performing arts professionals during this period (Spiro et al., 2021). Five participants in the present study (M = 1, S = 4) reported that this period had provided an opportunity for their bodies to recover from the strains of playing, supporting evidence of the presence of performance-related physical problems among orchestral musicians (Kok et al., 2016): “Physically I'm recovering from the strains of aches and pains, so that side of things is better, yeah” (S1). Seven participants (M = 4, S = 3) mentioned the positive effects of lockdown on family life, described by a parent of younger children as follows: “We have had an amazing opportunity to be together as a family, I have seen milestones… I've been able to see my son literally change on a daily basis because I'm at home all the time” (M12), and a parent of older children commented: “My kids are going to leave home, both of them will have left within three years, and I'm actually sitting here going ‘god, I'm really treasuring this time to be here with them, on a much more continuous basis”' (S4), supporting previous evidence that portfolio musicians can find it difficult to achieve a satisfactory work/family balance and that this can be a source of stress in their lives (Teague and Smith, 2015). Eight participants (M = 5, S = 3) had had *new insights* (sub-theme 5.3), which included: “It's been like a real sort of period of, of, dunno, just sort of re-, re-igniting certainly that love of music for music's sake… a bit of a kick up the arse in terms of taking things for granted, for sure” (M10), supporting the suggestion that unplanned changes in one's work situation have the potential to make a positive impact on individuals (Zikic and Klehe, 2006).

Two additional themes were identified, which were not encompassed by the overarching themes. These were *being ill with the virus* and *awareness of others*. Half of the participants (M = 4, S = 8) thought that they had been ill and contracted the virus during March or the start of April 2020. None of them had been tested, and several participants described how they had continued working while they were ill and almost certainly contagious, supporting the suggestion that the UK's lockdown measures were put in place too late (Anderson et al., 2020): “I felt pretty dodgy, and I had a temperature, I went to work and then lost, both of us [her and partner] lost our taste…I was teaching one-to-one, and scraping reeds and giving reeds out to people, yeah, not thinking about everything. Big isn't it?” (M3). One participant described the freelancer's attitude toward working just before the lockdown was announced, illustrating the financial vulnerability of freelance musicians (Bartleet et al., 2019; Willis et al., 2019):

I remember sitting around a table, even a week before [the lockdown] at X studios, saying to Y “we shouldn't be here”… but nobody said anything… you know the saying that in any given orchestra the freelance players will always be more ill than people who they're depping for, it's because we, you know, as freelancers, you can't really afford to be ill, you go in… I did feel that compulsion to do, to carry on even against my better judgement, because it's, my income depends on it, and who was I to doubt the, the government advice? Wow, I haven't really thought about that. (S9)

The other additional theme, *awareness of others*, was present in nearly all of the interviews (M = 11, S = 12) in which, in spite of the anxiety and uncertainty in their own lives, participants expressed concern for others who might be in a worse position than themselves. The concern of older participants for younger, less established musicians was a recurrent theme (M = 3, S = 9), expressed in worries that younger musicians may be financially vulnerable, had not yet had a chance to fulfill their professional dreams and ambitions, the difficulty of coping with a young family during this time, and a desire to help: “I'm, I'm extremely anxious for the younger generation… it's all very well having 25-plus years' experience in the profession, but they're at the very beginning of all this” (S6). Concern for others was also identified in Spiro et al.'s (2021) study of performing arts professionals' wellbeing during lockdown. The awareness and empathy for others exhibited by the participants in the present study may reflect the acute listening and communication skills that are an essential part of the orchestral musician's tool-kit, enabling them to carry out their everyday work of making music together (Myers and White, 2012).

## Discussion

This qualitative interview study investigating the impact of COVID-19 on the lives of established mid-career and seasoned self-employed freelance orchestral musicians found that all 24 participants had experienced the sudden loss of a much-loved and previously successful performing career, were concerned about their individual future careers and the future of the music profession, and were nearly all worried about money. In addition, it found that mid-career participants reported greater distress, worry about money, and confusion over identity and future careers than the seasoned participants. The mid-career participants appeared to be less motivated to practice, less engaged in collaborative music making, getting less pleasure from the music making that they were doing and less emotionally robust than their seasoned colleagues. These factors come together to create a concerning picture of a population of previously successful mid-career musicians in crisis. The findings of the study will now be examined with reference to lifespan models of a musician's career (Manturzewska, 1990; Bennett and Hennekam, 2018; López-Íñiguez and Bennett, 2020), the PERMA model of wellbeing (Seligman, 2012), and the concept of *resilience* (Bonanno et al., 2011) in order to gain a greater understanding of the differences between the experiences of mid-career and seasoned self-employed freelance orchestral musicians at this time.

Lifespan models of professional musicians' working lives (Manturzewska, 1990; Bennett and Hennekam, 2018; López-Íñiguez and Bennett, 2020) suggest that, while professional musicians focus on their own peak performing achievements during the mid-career stage (approximately ages 30–45), they focus to a greater extent in the late-career stage (approximately ages 55 and older) on teaching, passing on skills, social responsibility, and acknowledging their achievements. These stages in musician's lifespan models are compatible with the adult stages in Erikson's psychosocial theory of personality development (Erikson, 1964), in which Young Adulthood (approximately ages 25–40) is described as a time of forming loving relationships, consolidating goals and developing a meaningful career, and Middle Adulthood (approximately ages 40–65) is described as a period marked by a shift in one's perspective on time, feelings of accomplishment and a focus toward creating or nurturing things that will outlast the individual and benefit others. In terms of understanding our mid-career participants' identity confusion and distress, lifespan models suggest that, during the mid-career period, having aspired and worked toward their careers as professional musicians from a very early age (McPherson et al., 2012), they should have been at the peak of their career achievements (Gembris et al., 2018). The COVID-19 pandemic brought what could have been expected to be a period of career consolidation and peak playing achievement to a sudden, dramatic stop, leaving these highly motivated and dedicated musicians largely unable to work, in financial crisis and confusion about their future careers and also, for those with young families, dealing with full-time childcare. In contrast, the seasoned musicians who took part in our study were able to look back from a position of relative financial stability on long and successful performing careers, and many of them were already highly focused on instrumental teaching.

Several previous studies have examined musicians' wellbeing through the lens of the PERMA model (Ascenso et al., 2017, 2018), whereby wellbeing is conceptualized as comprising five components (Seligman, 2012): (1) Positive Emotion—the affective part of feeling good, (2) Engagement—deep psychological connection and absorption in an activity, (3) Relationships—the presence of social connections, (4) Meaning—a sense of purpose and sense that one's life matters, and (5) Achievement—a sense that internal and/or external goals are being achieved. An examination of the impact of COVID-19 on each of these aspects of wellbeing reveals that mid-career participants were experiencing less Positive Emotion than seasoned participants: the mid-career participants were more anxious about money and were more distressed and confused about their future careers than the generally more financially robust seasoned participants. Furthermore, those mid-career participants with young children were also managing the additional stress of childcare and home-schooling. Mid-career participants were less motivated to practice, less involved in and less satisfied by any collaborative playing that they were doing and had lower levels of Engagement in music making during this time than the seasoned participants. The practicalities of managing full-time childcare responsibilities may also have contributed to mid-career participants' lower levels of Engagement in music making, as described by Spiro et al. (2021) in their recent survey of performing arts professionals during lockdown. In terms of Relationships, both mid-career and seasoned participants were greatly missing colleagues and the social aspects of playing, although the mid-career participants with young children also had the additional stress of managing intense family relationships (including childcare and home-schooling) during this period. The seasoned participants' sense of their lives having Meaning did not appear to be challenged by this period; many of them were focused on teaching and expanding their musical skills and approaches. In contrast, the mid-career participants experienced this period as threatening to their identity as a musician, and had started to question the value and meaning of their musical careers. The seasoned participants were able to look back on their Achievements over the course of a long successful career; by contrast, the mid-career participants expressed frustration at being stopped in the middle of enjoyable careers that they had long worked toward.

The lifespan and PERMA models provide some insight into factors that may be contributing to the greater distress of mid-career musicians in comparison with seasoned musicians. Recent studies have found that levels of psychological distress during COVID-19 are higher in young adults and those under 50 than in older adults (Conversano et al., 2020; Glowacz and Schmits, 2020), suggesting that older people appear to be demonstrating greater resilience at this time. Older people's resilience, defined as the ability to maintain relatively stable, healthy levels of psychological and physical functioning when exposed to a potentially highly disruptive and adverse event (Bonanno et al., 2011) may be explained by older adults' greater cumulative life experience which, according to the strength and vulnerability integration model (SAVI; Charles, 2010), enables them to “navigate their worlds more successfully than younger adults” (Charles, 2010, p. 1073). This ability to develop and use coping strategies more effectively may also allow older adults to reach a higher threshold before perceiving a negative event as a stressor (Neubauer et al., 2019). Thus the greater resilience of seasoned players may also help to account for the findings of the present study. In addition, when one considers the research showing that musicians tend to be passionate about their performing careers (Bonneville-Roussy and Vallerand, 2020) and are deeply invested in and identified with their work (Dobson, 2010), and that involuntary career transitions are seen as a major threat to wellbeing in this population (Maitlis, 2009; Oakland et al., 2012; Hennekam and Bennett, 2016), it is clear that mid-career musicians are currently extremely vulnerable.

The question then arises of what might be done to help mid-career musicians. At the time of writing, it looks likely that the performing arts sector will be one of the last to return to work and that the upcoming period will remain very uncertain for self-employed musicians. Post-traumatic Growth (PTG) is a term used to describe the possibility of transformative positive growth and change as a response to a struggle with great adversity, however traumatic and unwanted the changes may be (Tedeschi and Calhoun, 2004). According to Maitlis (2020) there are two main steps in PTG. The first step involves managing the high levels of distress and emotional arousal that generally accompany great upset and can impair cognitive capacity (Van der Kolk and Fisher, 1995). Once an individual is able to function at a lower level of emotional arousal, the second step, *sensemaking*—defined as “a meaning-making process through which people work to understand unexpected or confusing events” (Maitlis, 2020, p. 405) —can begin. Sensemaking happens through sharing the story or narrative of events with trusted others who provide what Maitlis calls *attentive companionship*, which helps to support and facilitate growth-orientated sensemaking of a new reality. Maitlis emphasizes that this attentive companionship does not need to come from experts; the most important thing is for the “companions” to have “patience, empathy and the capacity to listen well” (Maitlis, 2020, p. 407). Given the growing evidence that many freelance musicians are currently in crisis (ENCORE Musicians, 2020; Musicians' Union (MU), 2020), musicians' organizations might do well to start training attentive companions who will be able to help distressed and confused musicians on the path toward PTG. Moreover, given that the theme of “awareness of others” was identified in almost all the interviews, that acute listening skills and the ability to build rapport and collegiality are essential components of the freelance musician's skill-set, and that several seasoned musicians described wanting to help their younger colleagues, it is possible that seasoned musicians might be the ideal people to be trained as attentive companions for the explicit purpose of helping younger musicians to navigate their way through this period. This is supported by the description by one of our mid-career participants of how helpful it had been to attend online meetings set up by musicians' organizations, and to talk with and be supported by other musicians. Recent research has suggested that effective strategies for cultivating resilience during this time of the COVID-19 pandemic include making meaning, building distress tolerance, and increasing social connectedness (Polizzi et al., 2020), all of which are encompassed by this model of PTG. Although the process of setting up the structures to train attentive companions at this time is likely to be complex given the requirement for individuals to maintain social distancing, online training should be feasible, as this is currently offered by many professional and volunteer psychological support services (Bell et al., 2020; Samaritans, 2021).

The limitations of this qualitative study include the carrying-out of interviews toward the start of the pandemic, during May and June 2020, providing a snapshot of musicians' experiences only during the first UK lockdown. Since then the situation has changed, and is continuing to do so, with lockdown restrictions of varying severity being imposed dynamically in response to regional changes in the number of cases of COVID-19. A follow-up study is planned for the summer of 2021, to investigate the impact of COVID-19 on these participants over a longer time scale. Although using Zoom as an alternative to meeting face-to-face allowed participants to be interviewed in the comfort and safety of their own homes, it did have the inherent disadvantages of occasional problems with the internet connection and the difficulty of maintaining eye contact (although this was generally not a problem as both interviewer and interviewee tended to look directly at the computer screen and thus straight into the computer camera, creating an experience of eye contact). The potential difficulty of creating private space for the duration of the interview (Marhefka et al., 2020) was not an issue in any of the interviews, despite the fact that many participants were at home with family members. The first author, who carried out the interviews, had previously worked as a freelance self-employed musician. The research benefitted from her insider's perspective, which enabled her to establish rapport and ask relevant questions with ease, while the second author (also a former freelance self-employed musician, albeit a singer rather than an orchestral player) provided a source of triangulation for ensuring the credibility and trustworthiness of the analyses. The first author was aware of the potentially sensitive and emotional nature of the subject matter and, while she aimed to provide an opportunity for participants to paint a realistic picture of their experiences during this time, she also tried to ensure that participants did not experience increased levels of distress (Jowett, 2020). The semi-structured format of the interviews provided the flexibility to do this, and many participants mentioned at the end of the interviews that they had found the opportunity to reflect and think things through useful.

In conclusion, the findings of the current study suggest that the COVID-19 pandemic has had a severe impact on the lives of classical self-employed freelance orchestral musicians, and especially so for mid-career musicians who appear to be emotionally and financially more vulnerable and to demonstrate less resilience than their older, more seasoned colleagues. However, the findings also reveal that, even in these times of great stress, musicians are aware of and feel concern for others who may be in a worse position than themselves. Thus it is possible, based on Maitlis' model of sensemaking in PTG, that seasoned musicians could be ideal candidates to be trained as attentive companions so as to help younger musicians to navigate their way through this challenging time.

## Data Availability Statement

The raw data supporting the conclusions of this article will be made available by the authors, without undue reservation.

## Ethics Statement

The studies involving human participants were reviewed and approved by The Ethics Committee at the Royal Northern College of Music, Manchester. The participants provided their written informed consent to participate in this study.

## Author Contributions

SC and JG designed the study and procedures and analyzed the data. SC carried out the interviews. SC drafted the manuscript with support from JG in all sections. Both authors approved the submitted version.

## Conflict of Interest

The authors declare that the research was conducted in the absence of any commercial or financial relationships that could be construed as a potential conflict of interest.
