# Supplementary material for: The Experiences of Mid-career and Seasoned Orchestral Musicians in the UK During the First COVID-19 Lockdown
Source: Front Psychol. 2021 Apr 9;12:645967. doi: 10.3389/fpsyg.2021.645967 (PMC8062715; doi:10.3389/fpsyg.2021.645967)
Supplement: Supplementary file 1 [file Table_1.DOCX]

**SUPPLEMENTARY MATERIAL**

**APPENDIX A: INTERVIEW GUIDELINES FOR SEMI-STRUCTURED INTERVIEW**

1. Please could you give a short autobiography of your career as an orchestral musician?
2. Can you say something about the way in which COVID-19 has impacted on your life?

Prompts:

1. Have you had work cancelled?
2. What impact has this had on your finances?
3. How are you feeling about and managing all of this?
4. Have you applied for any of the government schemes/other organisations (HMUK) for financial support?
5. Are you currently practising?
6. Are you playing with other musicians? If so, what and how are you working?
7. What are you missing?
8. How are you managing all the uncertainty surrounding this time?
9. Could you say something about where your mood is?
10. What are your thoughts about the future?
11. What do you do to help yourself keep going?
12. Have you had COVID-19?
13. Could you say something about your use of social media at the moment?
14. Are there good things that are coming out of this period for you?
15. Is there anything else you’d like to add?
